# Dengue knowledge, attitudes and practices and their impact on community-based vector control in rural Cambodia

**DOI:** 10.1371/journal.pntd.0006268

**Published:** 2018-02-16

**Authors:** Emmanuelle Kumaran, Dyna Doum, Vanney Keo, Ly Sokha, BunLeng Sam, Vibol Chan, Neal Alexander, John Bradley, Marco Liverani, Didot Budi Prasetyo, Agus Rachmat, Sergio Lopes, Jeffrey Hii, Leang Rithea, Muhammad Shafique, John Hustedt

**Affiliations:** 1 Technical Department, Malaria Consortium, Phnom Penh, Cambodia; 2 Department of Infectious Disease Epidemiology, London School of Hygiene and Tropical Medicine, Keppel Street, London, United Kingdom; 3 National Dengue Control Program, National Center of Parasitology, Entomology and Malaria Control, Ministry of Health, Phnom Penh, Cambodia; 4 Malaria and other Vector-borne and Parasitic diseases Office of the WHO Representative in Cambodia, World Health Organization, Phnom Penh, Cambodia; 5 Department of Global Health and Development, London School of Hygiene and Tropical Medicine, Keppel Street, London, United Kingdom; 6 Entomology Unit, US Naval Medical Research Unit-2, Phnom Penh, Cambodia; 7 Data Analyst Unit, US Naval Medical Research Unit-2, Phnom Penh, Cambodia; Oxford University Clinical Research Unit, VIET NAM

## Abstract

**Background:**

Globally there are an estimated 390 million dengue infections per year, of which 96 million are clinically apparent. In Cambodia, estimates suggest as many as 185,850 cases annually. The World Health Organization global strategy for dengue prevention aims to reduce mortality rates by 50% and morbidity by 25% by 2020. The adoption of integrated vector management approach using community-based methods tailored to the local context is one of the recommended strategies to achieve these objectives. Understanding local knowledge, attitudes and practices is therefore essential to designing suitable strategies to fit each local context.

**Methods and findings:**

A Knowledge, Attitudes and Practices survey in 600 randomly chosen households was administered in 30 villages in Kampong Cham which is one of the most populated provinces of Cambodia. KAP surveys were administered to a sub-sample of households where an entomology survey was conducted (1200 households), during which *Aedes* larval/pupae and adult female *Aedes* mosquito densities were recorded. Participants had high levels of knowledge regarding the transmission of dengue, *Aedes* breeding, and biting prevention methods; the majority of participants believed they were at risk and that dengue transmission is preventable. However, self-reported vector control practices did not match observed practices recorded in our surveys. No correlation was found between knowledge and observed practices either.

**Conclusion:**

An education campaign regarding dengue prevention in this setting with high knowledge levels is unlikely to have any significant effect on practices unless it is incorporated in a more comprehensive strategy for behavioural change, such a COMBI method, which includes behavioural models as well as communication and marketing theory and practice.

**Trial registration:**

ISRCTN85307778.

## Author summary

The global incidence of dengue has grown dramatically over the last few decades and has become the most rapidly spreading mosquito-borne disease. To date, there is no specific treatment. A vaccine came on the market in 2015, but it will be several years before it becomes widely available and its efficacy is limited. Therefore vector control is the most important means of dengue prevention at the current time. The World Health Organization recommends the adoption of an integrated vector management approach using community-based methods tailored to the local context. In order to design appropriate strategies, it is essential to understand local knowledge, attitudes and practices regarding dengue vector control. We conducted a survey in the Cambodian province of Kampong Cham, to investigate the local knowledge levels as well as self-reported vector control practices and observed practices. We found a high knowledge of dengue transmission, and *Aedes* breeding and biting prevention methods. However, no correlation was found between self-reported vector control practices and observed practices. Additionally, knowledge levels did not correlate with actual vector control practices.

## Introduction

With up to 3.9 billion people in 128 countries at risk of the disease, dengue affects most of the world’s tropical and sub-tropical regions and has become the most rapidly spreading mosquito-borne viral disease[1,2]. There are an estimated 390 million infections per year, of which 96 million are clinically apparent [3].

There is currently no cure available for dengue. In 2015, the first ever dengue vaccine, Dengvaxia (Sanofi-Pasteur), came on the market despite having 60% efficacy and inducing very low protection against DENV-2[4,5]. It will also likely be several years before the vaccine is made available in low-income countries such as Cambodia. Due to the lack of a readily available vaccine or therapeutics, vector control is the only means of dengue prevention.

There are an estimated 185,850 dengue cases in Cambodia annually [6,7]. Since the early 1990s, the primary means of vector control by the Cambodian National Dengue Control Program (NDCP) has been the use of the organophosphorous larvicide temephos, under the trade name Abate, applied in water storage containers [8]. However, evidence of *Ae*. *aegypti* resistance to temephos has been found in Cambodia and in other parts of Southeast Asia since 2001 [9–13]. Thus, it is clear that alternative vector control strategies are needed. Previous studies have demonstrated the most effective vector control approaches used community-based methods tailored to the local context [14–17]. In order to develop a successful strategy, it is therefore crucial to gain an understanding of current knowledge and practices regarding vector control and dengue fever in the communities.

This study aimed to evaluate people’s knowledge of the dengue vector and control methods, their perceived risk of dengue fever, and to compare reported versus observed household practices. Knowledge, Attitudes and Practices (KAP), and entomology surveys were conducted to ascertain these measures and respective relations.

## Materials and methods

### Study context

The study took place in Kampong Cham, a large and populous province located in central Cambodia, chosen for its high dengue incidence [18]. Being a largely rural province, the main occupation is agriculture, and it has literacy rates of 74.8% for women and 81.3% for men[19]. The data were collected as part of a larger cluster randomized trial set up to evaluate the effect of placing guppy fish and WHO-approved insect growth regulators in household water containers on adult female *Aedes aegypti* mosquito densities. The detailed protocol can be found in a previously published manuscript[20]. Briefly, 30 clusters containing one or more villages were randomly assigned to three different arms. Arm 1 received guppy fish in large water containers, the insect growth regulator in smaller containers and communication activities to promote community engagement and uptake, based on the Communication and Behaviour Impact (COMBI) approach (20). Arm 2 received only guppy fish and COMBI activities. The third arm, the control, received only standard vector control activities from the Ministry of Health which includes outbreak response in villages with three or more cases.

Prior to the trial, a baseline KAP survey was administered to one participant from each of 600 randomly selected household (20 HHs per cluster in 30 clusters). Additionally, baseline entomology surveys were conducted in 1200 households (40 per cluster in 30 clusters).

### Knowledge, attitudes and practices survey

The KAP survey was administered to participants in September 2015, prior to the start of the interventions. The survey questionnaire (S1 Survey) was formulated using data previously collected as part of focus group discussions and in-depth interviews in a neighbouring community. The survey was piloted in a village in Kampong Cham not involved with the study (20km from study site) to assess comprehensibility and refine the formulation of questions. Following written informed consent, the questionnaire was administered face-to-face in Cambodian language to all participants, and included both structured and open-ended questions about participants’ knowledge of the dengue vector, the vector’s breeding sites and breeding prevention methods as well as dengue symptoms and treatment. The KAP survey also measured household wealth using a set of questions on asset ownership based on those included in the national Demographic and Health Survey (DHS) and adapted to the local context [21]. This information was used to generate a measure of socio-economic status (SES) which is further described below. A detailed description of the methods for both the entomology and KAP survey is provided in a previously published paper [20].

### Entomology survey

The baseline entomology surveys were done at the same time as the KAP surveys. The methodology used was based on the WHO guidelines for entomological collections [2]. All containers in surveyed households were inspected. Larvae and pupae collection in containers larger that 50L was conducted using the *five sweep method* [22]. The contents of smaller containers were emptied out into the sweep net. Resting adults were caught with a portable aspirator (Camtech, Phnom Penh, Cambodia): the walls in bedrooms and living spaces were aspirated in a clockwise manner up and down the wall for 10 minutes per house.

### Data management and statistical analysis

Data were double entered into EpiData (EpidData Association Denmark) by an experienced external data entry team. Textual data collected in open-ended questions was translated into English and coded. All analysis was done using the statistical analysis software Stata 14.1.

Principal component analysis (PCA) using durable asset ownership was performed in order to classify participants into socio-economic quintiles [23,24]. A descriptive analysis of the different asset variables was carried out to determine their frequency and standard deviation. Variables with very low counts (<0.01%) were excluded from further analysis. A co-variance matrix was generated for the PCA analysis as all the variables were standardized to the same unit (binary yes = 1/no = 0). From this matrix a PCA analysis was performed using the Stata PCA command. The results of this analysis were used to generate a wealth score, which was then used to classify the participants into socio-economic quintiles.

In order to determine the factors influencing knowledge, Chi squared tests as well as univariate followed by multivariate logistic regressions were performed. The multivariate regression models were built using a backward elimination approach. Variables found to be have a p-value<0.1 were kept in the model.

Linear regression was used to analyse the relation between knowledge of mosquito breeding levels and reported behaviours and their observed behaviours. Knowledge of mosquito breeding was defined as the mean number of miscellaneous mosquito breeding containers identified per cluster. A negative binomial regression was used to analyse relationship between adult mosquito densities and reported behaviours.

Where appropriate, robust standard errors were used to account for intra-cluster correlation.

### Ethics

This study received approval from the Cambodian National Ethics Committee for Health Research on 9 October 2014 (reference number 0285). Additionally ethics approval was also received from the London School of Hygiene and Tropical Medicine ethics review board (reference number 10704). The trial was registered with the International Standard Randomized Controlled Trial Number Register: ISRCTN85307778.

## Results

### Participant demographics

Of the 600 participants who were administered the KAP survey, the majority were female (77.8%), most likely because the survey was conducted during the day when the men are out working (Table 1). The ages of the participants ranged from 17 to 88 years with the mean age being 44. One per cent of participants were less than 20 years of age. In Cambodia it is common for people to marry early. Indeed, the most recent Demographic and Health Survey (2014) showed that 4.4% of women were married by the age of 15 and 45% by the age of 20 [19]. The highest level of education attained for most of the respondents was primary school (59.2%). Near 20% of participants had no formal education or had received the alternative education and training during the Khmer Rouge rule. The primary occupation of people surveyed was farming (70.2%), followed by manual labour (12%).

**Table 1:** Demographic information about participants surveyed.

Characteristics	% (N = 600)	Median (range)
**Sex**		-
Female	77.8 (467)	-
Male	22.2 (133)	-
**Age (range, median)**		43 (17–88)
15–34 (17–34,28)	28.2 (169)	28 (17–34)
35–54 (35–54,45)	47.3 (284)	44.5 (35–54)
55+ (55–88, 62)	24.5 (147)	62 (55–88)
**Education**		-
None	19.8 (119)	-
1–6 years	59.2 (355)	-
>6 years	21 (126)	-
**Occupation**		-
Unemployed/stay at home	7.5 (45)	-
Farmer	70.2 (421)	-
Office staff	2.2 (13)	-
Laborer	12 (72)	-
Small tradesmen	7.2 (43)	-
Other	1 (6)	-

### Knowledge

Amongst the survey participants, knowledge regarding dengue transmission, prevention methods and symptoms was high. The vast majority of people (96.7%) were able to identify mosquitoes as being the dengue vector (Table 2). The majority of people surveyed were also correctly able to identify the dengue mosquito biting times, although, 17.8% of participants believed that the dengue vector bites at night. The majority of respondents (95.5%) were able to correctly identify at least one breeding site. Water storage jars, coconut shells/cans were the most commonly cited breeding sites (85.1% and 78.6% respectively).

**Table 2:** Knowledge of dengue transmission, prevention practice and symptoms.

	n (N = 600)	% (95% C.I.)
**How is dengue transmitted?**		
Mosquito	581	96.7 (94.3–98.1)
**When do dengue mosquitoes most often bite?**		
Day	425	74 (69.6–78)
Night	103	17.8 (14.5–21.6)
Don’t know	53	8.2 (5.4–12.4)
**Where can the dengue mosquito breed?**		
Don’t Know	10	2.3 (0.9–6.2)
Water storage jars	499	85.1 (81.5–88.1)
Coconut shells/cans	468	78.6 (71.4–84.4)
Tyres	369	63.7 (58–69)
Anything with water around the house	309	53.4 (48.7–58.1)
Cement baths	190	32.1 (28.6–35.9)
Ant traps	114	18.0 (13.4–23.9)
Knows 1 or more breeding sites	575	95.5 (93.1–97.1)
**How can you prevent mosquitoes from breeding?**		
Don’t Know	14	2.7 (1.5–4.8)
Use Abate	452	73.1 (68.9–76.9)
Changing stored water frequently	317	53.7 (47.3–60)
Turn containers upside down	252	40.8 (36.4–45.3)
Put fish in water jars	133	22.2 (17.3–28.2)
Put lids on water jars	115	20.1 (15.8–25.3)
Spraying insecticide	78	13.5 (10.0–17.8)
Knows 1 or more prevention methods	569	93.9 (90.6–96.1)
Knows 1 or more prevention methods other than Abate	493	81.33(75.5–86)
**How can you prevent mosquitoes from biting you or your family?**		
Don’t know	6	1.2 (0.3–4.3)
Use mosquito net during the day	350	59 (54.4–63.4)
Wear long sleeves/ long pants	292	49.2 (45.6–52.8)
Use mosquito coils during the day	286	46.1 (39–53.4)
Keep household environment clean	164	27 (21.8–32.9)
Burn/Bury coconut shells	160	25.6 (21.3–30.5)
Use fan	122	21.2 (14.6–29.9)
Cut down bushes near the house	102	15.6 (12.4–19.4)
Have children play far from mosquito breeding sites	93	16.5 (12.5–21.3)
Keep cloths tidy	54	8.1 (5.7–11.3)
Electricity trap	36	6.2 (4.4–8.8)
Use mosquito repellent	40	6.5 (4.7–8.8)
Knows 1 or more mosquito bite prevention methods.	562	94.1 (91.7–95.9)
**What are the symptoms of dengue?**		
Don’t know	32	5.1 (3.5–7.4)
Fever	553	92.1 (89.3–94.3)
Somnolence	349	58.5 (54.5–62.4)
Rash	302	49.2 (44.2–54.3)
Headache	60	9.9 (7.5–13.1)
Nausea/Vomiting	51	8.3 (5.9–11.5)
Bleeding	42	6.7 (4.4–10.1)
Shock	26	4.8 (2.8–8.1)
Aches and Pains/Body pain	14	2.4 (1.4–4.2)
Muscular Pain	8	1.7 (0.7–4.4)
Know 3 or more symptoms	264	42.7 (39.2–46.4)

When asked about mosquito breeding prevention methods, 93.9% of participants knew of at least one mosquito breeding prevention method (Table 2). Indeed, in response to an open-ended question about the types of breeding prevention methods, the most commonly cited method was the use of Abate (73% of respondents); 12.6% of respondents only cited Abate, while 53.7% also mentioned changing water frequently in storage jars. Almost all participants (94.1%) knew at least one mosquito-bite prevention method. The use of nets during the day was the most commonly cited method (59%). Certain methods were strongly associated with participant’s SES level. For example, 45% (95% CI 33.24–56.59) of respondents from the highest SES quintile mentioned the use of fans compared with only 6% (95% CI 2.56–15.03) from the lowest quintile.

Knowledge of dengue symptoms was much less common (Table 2). In order to distinguish dengue from other febrile illnesses, WHO recommends looking for the presence of high fever and at least two other symptoms such as rash, aches and pains, mucosal bleeding and nausea/vomiting [2]. Although 92.1% of people mentioned fever as one of the symptoms of dengue, only 42.7% could name three or more symptoms.

Education was the main predictor of knowledge amongst participants (Table 3). After adjusting for age, respondents with at least 6 years of education had almost seven times the odds of knowing mosquitoes are the vector of dengue and four times the odds of being able to name at least one mosquito breeding site. Gender was found to be a strong predictor of knowledge regarding dengue symptoms: after adjusting for education and socio-economic status, female participants had a 63% higher odds of being able to name three or more dengue symptoms.

**Table 3:** Predictive factors of knowledge regarding dengue transmission, prevention practices and symptoms.

	Univariate			Multivariate		
Variable	Crude OR	95% C.I.	p-value	Adjusted OR	95% C.I.	p-value
**Dengue is transmitted by mosquitoes**						
Female	0.81	0.31–2.13	0.659	-	-	-
Male	1.00	-	-	-	-	-
Age < 35 years	4.38	0.45–42.54	0.192	3.14	0.42–23.44	0.254
Age 35–54	1.00	-	-	1.00	-	-
Age > 54 years	0.29	0.14–0.61	0.002	0.45	0.22–0.89	0.023
>1 year of eduction	1.00	-	-	1.00	-	-
1–6 years of education	5.96	2.36–15.08	p<0.001	4.13	1.63–10.48	0.004
> 6 years of education	14.55	1.70–124.86	0.016	6.98	1.06–45.98	0.044
Low SES	1.00	-	-	-	-	-
Middle SES	0.47	0.13–1.73	0.248	-	-	-
Highest SES	4.01	0.36–45.19	0.250	-	-	-
**Knows at least one mosquito breeding site**						
Female	0.55	0.20–1.54	0.245	-	-	-
Male	1.00	-	-	-	-	-
Age < 35 years	1.33	0.31–5.76	0.691	1.05	0.21–5.29	0.949
Age 35–54	1.00	-	-	1.00	-	-
Age > 54 years	0.38	0.23–0.63	p<0.001	0.57	0.33–0.99	0.048
>1 year of eduction	1.00	-	-	1.00	-	-
1–6 years of education	5.14	1.91–13.84	0.002	4.26	1.74–10.44	0.003
> 6 years of education	5.68	1.39–23.28	0.017	4.34	0.94–19.95	0.059
Low SES	1.00	-	-	-	-	-
Middle SES	0.60	0.21–1.69	0.318	-	-	-
Highest SES	3.37	0.33–33.97	0.291	-	-	-
**Believe dengue mosquitoes bite most often at night.**						
Female	0.62	0.41–0.95	0.031	0.65	0.42–1.01	0.056
Male	1.00	-	-	-	-	-
Age < 35 years	1.38	0.88–2.46	0.155	-	-	-
Age 35–54	1.00	-	-	1.00	-	-
Age > 54 years	1.31	0.70–2.46	0.385	-	-	-
>1 year of eduction	1.00	-	-	1.00	-	-
1–6 years of education	0.76	0.43–1.36	0.345	-	-	-
> 6 years of education	0.54	0.24–1.20	0.124	-	-	-
Low SES	1.00	-	-	-	-	-
Middle SES	1.01	0.60–1.70	0.958	0.99	0.59–1.68	0.979
Highest SES	0.40	0.21–0.78	0.009	0.40	0.20–0.79	0.010
**Knows at least one mosquito bite prevention method**						
Female	1.47	0.70–3.11	0.300	-	-	-
Male	1.00	-	-	-	-	-
Age < 35 years	1.77	0.73–4.30	0.197	-	-	-
Age 35–54	1.00	-	-	1.00	-	-
Age > 54 years	0.56	0.30–1.05	0.071	-	-	-
>1 year of eduction	1.00	-	-	1.00	-	-
1–6 years of education	2.88	1.50–5.54	0.002	2.45	1.27–4.76	0.010
> 6 years of education	13.53	1.46–125.30	0.023	10.22	1.07–98.02	0.044
Low SES	1.00	-	-	-	-	-
Middle SES	0.93	0.34–2.56	0.892	0.89	0.31–2.59	0.825
Highest SES	11.49	1.15–114.16	0.038	8.32	0.80–86.60	0.075
**Knows at least three dengue symptoms.**						
Female	1.48	0.93–2.34	0.094	1.63	1.01–2.65	0.046
Male	1.00	-	-	-	-	-
Age < 35 years	1.00	0.67–1.48	0.992	-	-	-
Age 35–54	1.00	-	-	1.00	-	-
Age > 54 years	1.09	0.68–1.75	0.703	-	-	-
>1 year of eduction	1.00	-	-	1.00	-	-
1–6 years of education	1.07	0.70–1.63	0.747	1.01	0.65–1.58	0.954
> 6 years of education	1.70	1.15–2.51	0.010	1.59	1.08–2.34	0.019
Low SES	1.00	-	-	-	-	-
Middle SES	1.27	0.62–2.62	0.497	1.28	0.60–2.73	0.511
Highest SES	2.47	1.32–4.65	0.006	2.31	1.20–4.42	0.014

### Health seeking behaviour

If the participants or a member of their family developed a fever, just over 32.2% of them would seek care from a health facility or private provider (Table 4). The majority of people would self medicate either by getting drugs from the pharmacy (i.e. paracetalmol) (18.6%) or using cold compresses (26.4%). Four percent of respondents mentioned they would practice “scratching”, a traditional practice that involves scratching the body with a coin to cure illness. Men were found to be more likely to seek medical attention from a health facility or private provider. Amongst the men surveyed, 43.7% said they would seek care, compared to only 29% of women (χ^2^ test, P<0.01).

**Table 4:** Health seeking behaviour regarding fever.

	**n (N = 600)**	**% (95% C.I.)**
**If you or someone in your family has a fever, what would you do first?**		
Go to the Health Facility	120	20.32 (16.8–24.4)
Go to a Private provider	76	11.91 (8.8–15.9)
Go to a Community Health Worker	5	0.8 (0.2–2.6)
Get medication from the pharmacy	109	18.6 (15.7–21.9)
Wait for the fever to go away	21	3.4 (1.8–6.2)
Use cold compress	162	26.4 (22.4–30.8)
“Scratching”*	20	4.3 (1.9–9.4)
Don’t know	1	0.07 (0.01–0.59)
Other	86	14.17(10.49–18.86)
	**Time (days)**	**95% C.I.**
**Average time participants would wait before seeking care**		
Lowest SES quintile	2.4	2.1–2.6
Middle SES quintile	2.	1.8–2.2
Highest SES quintile	1.8	1.6–2

* Scratching: traditional remedy where a practitioner/healer will rub a coin on the back, neck, arms and upper chest of the patient

Linear regression analysis revealed strong evidence of a negative correlation between a person’s SES and the amount of time waited before seeking care if they or a member of their family gets a fever (Coeff: -0.08, 95%CI: -0.12–-0.05, P<0.001). People in the lowest SES quintile stated that they would wait on average 2.35 (95% CI: 2.13–2.56) days whereas respondents in the highest SES quintile would wait 1.80 days (95% CI: 1.61–1.99) (Table 4).

### Attitude towards dengue

Almost all participants surveyed believed they are at risk of getting dengue (97.5%) and the majority also believed that the disease can be prevented (78%) (Table 5). No significant correlation was found between risk awareness and socio-cultural variables such as education, SES, gender, occupation and age.

**Table 5:** Attitude towards dengue fever.

	n (N = 600)	% (95% C.I.)
**Are you at risk of getting dengue?**		
Yes	583	97.5 (94.7–98.8)
No	6	1 (0.4–2.4)
Don’t Know	11	1.5 (0.5–4.9)
**Can dengue be prevented?**		
Yes	461	77.8 (74.1–81.1)
No	84	13.4 (10.6–16.9)
Don’t know	54	8.8 (6.2–12.4)

### From self-reported to observed practices

The effect of knowledge that miscellaneous containers such as soda cans, coconut shells and tyres are breeding sites on observed practices was assessed (Table 6). It was found that the mean levels of knowledge per cluster had no effect on the proportion of households found to have such containers. Furthermore, no correlation was found between village knowledge levels of miscellaneous containers serving as breeding sites and proportion of households found to have pupae/larvae in miscellaneous containers (Table 6).

**Table 6:** Regression and negative binomial regression analysis of cluster level knowledge, self-reported practices and observed practices.

	Regression Coefficient	Standard Error	P-value	R-squared
**Proportion of households with miscellaneous containers**				
Mean cluster knowledge of miscellaneous containers as breeding sites	0.97	0.54	0.860	0.0011
Proportion of households who reported clearing miscellaneous containers	0.08	0.34	0.821	0.0019
**Proportion of households with *Aedes* larvae/pupae in miscellaneous containers**				
Mean cluster knowledge of miscellaneous containers as breeding sites	0.12	0.42	0.782	0.0028
Proportion of households who reported clearing miscellaneous containers	0.05	0.27	0.858	0.0012
**Mean number of resting female *Aedes* mosquitoes per household**				
Mean cluster knowledge of miscellaneous containers as breeding sites	-0.10	2.75	0.971	>0.001
Proportion of households who reported clearing miscellaneous containers	5.28	2.31	0.022	0.1064

Analysis was also performed on the proportion of households per village reporting disposing of miscellaneous containers and proportion of households found to have these containers. No correlation was found between these factors either. Nor was there any correlation between the levels of self-reported disposal of containers and the proportion of households with pupae/larvae in miscellaneous containers (Table 6). There was however a strong positive correlation between the proportion of households per village who reported clearing these containers and the mean number of resting female *Aedes aegypti* mosquitoes per household per village (coeff. 5.28, standard error 2.1, p-value 0.02) (Table 6). Interestingly, the higher the number of households reporting the practice per cluster, the higher the mean number of resting mosquitoes per household was found.

## Discussion

Results from the KAP survey revealed a high knowledge of dengue transmission and prevention amongst participants. Consistent with other KAP surveys done in dengue endemic regions, the surveyed population was able to correctly identify mosquitoes as the dengue vector [25–29]. Nevertheless, close to a fifth of respondents believed *Aedes* mosquitos most often bite at night, suggesting a possible confusion with *Anopheles* (the night-time biting malaria transmitting mosquito). A similar study in Laos, found that as many as 33% of respondents were unaware that malaria and dengue are different diseases [28]. This confusion is not surprising given the historically high prevalence of malaria in the Greater Mekong Subregion and the similarities in clinical symptoms. However, in recent years the prevalence of malaria in Cambodia has dropped significantly and is now concentrated in the forested border regions of the country [30]. Age was found to be a predictor of both breeding site knowledge and vector knowledge. Participants over 54 years of age were much less likely to answer these questions correctly. Although not significant, younger people (age<35) seemed to be better able to identify mosquitoes as the vector and name potential *Aedes* breeding sites. During the horrific Khmer Rouge rule, which destroyed the county, traditional education was not permitted and agricultural production was given priority over literacy training and the implementation of their reformed education system. Cambodia has made great strides in rebuilding itself, but this historical legacy may explain why the older generations are less well educated than the younger generations. The results also suggest that perhaps the previous dengue campaigns have been less successful at reaching older people.

Over 95% of villagers interviewed could correctly identify a mosquito-breeding site. In rural parts of Cambodia, only 7% of households have access to improved drinking water piped onto their premises [31]. It is therefore common practice for people to store water in large water storage jars (200–400 litres) outside their houses. However, these water jars are an ideal breeding site for *Aedes* mosquitoes and have been shown to constitute over 80% of the larval habitats [32]. Eighty-five percent of participants were able to correctly identify these jars as breeding sites and this was the most cited answer.

Awareness regarding breeding and bite prevention methods was also very high. As with knowledge on transmission, the main predictor was education. Indeed, participants with at least 6 years of education had over ten times the odds of knowing at least one bite prevention method. The main method of *Aedes* breeding prevention cited was Abate. Over 73.1% of participants mentioned Abate in open-ended questions and over 10% only mentioned Abate. NDCP temephos distribution programme has been running since the early 1990s[8]. This long running programme explains the high knowledge of Abate amongst the interviewees. It also potentially explains why participants were able to identify the large water jars as breeding sites as the larvicide bags are usually placed in these water containers during the Abate distribution campaigns.

Gender, education and SES quintiles were all found to be predictors of dengue symptom awareness. Women had 63% higher odds of being able to correctly identify three or more symptoms. This most likely reflects women’s roles as caretakers. People in the higher SES quintile were also found to have higher odds of correctly identifying dengue symptoms. This may be due to an increase in access to information, for instance through better access to health facilities and other information sources. However, as a whole knowledge regarding dengue symptoms was generally lower. Despite 92.1% of people mentioning fever as a symptom, only 42.7% of individuals surveyed were able to correctly identify at least three dengue symptoms. These results mirror results from other studies, where the awareness that fever is a dengue symptom is usually higher than for the other symptoms [26,28,29,33–37]. This lack of knowledge of symptoms could be due to the wide spectrum of clinical manifestations seen in patients. It is also possible that past educational campaigns have focused more on the prevention of disease, rather than symptoms and early care seeking. As with many diseases, dengue clinical outcomes are greatly improved with early diagnosis and treatment. It is therefore important for people to be able to correctly identify potential dengue cases early and seek care.

Although almost all participants believe they are at risk of catching dengue, when asked about their health seeking behaviour, only 32.2% would seek medical care (20.3% would go to the health facility and 11.9% to a private provider) if they or someone in their family developed a fever. This hesitance to access medical care could be explained by the lack of adequate services provided at health facilities [38]. Indeed, in addition to drug stock-outs, health centres and health posts often suffer from insufficient staffing [38]. Furthermore, out-of-pocket payments have been shown to represent 73.1% of expenditures on health services [38]. When asked how long the villagers would wait before seeking medical attention, the amount of time they would wait correlated strongly with their SES level. Thus suggesting that their financial situation had a strong impact on their choice to seek care.

Men were also more likely to seek medical care. Indeed 43.7% of men responded they would go to a health facility or private provider. Studies on the relationship between health-seeking behaviour and gender in this context are limited. However, in a study conducted in Vietnam, a gender bias to males amongst patients hospitalised for dengue was noted despite their finding that women were at higher risk of severe dengue [39]. A KAP study performed in Venezuela found that, regardless of gender, 85.7% of people would not seek immediate care for fever [40]. However where dengue was suspected, 63.8% would seek care immediately. It is likely that, had participants in the present study been asked what they would do if they suspected a dengue infection, the frequency of people claiming they would seek care might have been higher in both sexes. Also, given their role as caregivers, it is possible that women may be more reluctant to leave the household, unless the symptoms are severe, thus explaining the difference if health seeking behaviour observed between men and women. Indeed, in an ethnography study also undertaken in Kampong Cham, it was found that women stated using traditional remedies and pharmacy bought medication when their children fell ill, only seeking medical care if the illness persisted [41].

Consistently with several other studies, the high levels of awareness regarding transmission and prevention methods did not translate into practice[26,28,34,36,42]. No correlation was found between cluster knowledge levels regarding the disposal of miscellaneous containers and the actual number of these containers found in households. These miscellaneous containers such as cans, coconut shells and tires are potential breeding sites. By disposing of these containers, the villagers can reduce potential larval habitats around their houses. In addition, no correlation was found between village awareness levels regarding these containers and the proportion of households found to have larvae/pupae in miscellaneous containers. Waste management has become one of the most pressing problems in Cambodia [43]. In the rural parts of the country, the problem is much worse as the responsibility is often left to individuals to manage their waste through burning and burying [43]. This could explain why people’s knowledge did not seem to translate into practice.

It is also possible that the participants did not feel it was their responsibility to assume vector control measures. With the temephos distribution programme in place for such a long time, it is possible that people believe it is the government’s responsibility to control the mosquito populations. This was observed in Cuba and Thailand, where the population believed it was up to government institution to control mosquito populations [44,45][26]. For this reason they may be less likely to actually take vector control measure despite knowing of their benefits.

The long running temephos distribution programme in Cambodia may also have contributed to a false sense of security in the community. In a large cluster randomised trial in Nicaragua looking at the effect of community mobilisation on dengue prevention found that the presence of temephos in water storage containers was a risk factor for dengue infections [17]. In was hypothesised that in households where the pesticide was being used, participants were less likely to take physical vector control measures because they felt protected by the pesticide.

Surprisingly, clusters where high numbers of households reported clearing these containers were found to have higher numbers of resting female mosquitoes. Although a correlation was found with the adult form of the mosquito, no correlation between the number of households with larvae/pupae and the level of reported practices. This could be because miscellaneous containers represent a small proportion of *Aedes* breeding sites. Indeed, as previously mentioned the large water storage jars are believed to represent 80% of *Aedes* breeding sites[32]. Also, because of the higher numbers of mosquitoes, these households may also be aware the they should be clearing these containers. Similar results were found by Koenraadt el.al, where participants with higher levels of knowledge regarding *Aedes* breeding sites had more potential breeding on their property [26].

The correlation analysis of knowledge and self-reported practices on observed practices was done at a cluster level. Due to the method of data collection, it was impossible to do this analysis at an individual or household level. This limits the analysis of the data as the cluster level analysis may mask individual level correlations. This study found that knowledge levels regarding dengue transmission, symptom and vector control was very high. However, it is worth noting that Kampong Cham has a high incidence of dengue and as a result they may have had more interventions than in other provinces in the country. Also, because of this high incidence, several other dengue studies have taken place in Kampong Cham [8,18,46–49]. As a result, it is possible that the knowledge levels recorded in this study may be higher than the rest of the country.

### Conclusion

Knowledge regarding dengue transmission and prevention methods was very high amongst participants, of which education was the main predictor. Villagers with at least 6 years of education had higher odds of being able to identify the *Aedes* biting times, biting prevention methods as well as *Aedes* breeding prevention methods. Although the results suggest knowledge regarding vector control measures was high, this did not translate into practice. In this setting with such high knowledge levels, an educational campaign is unlikely to have any real impact on practices. Instead a sustained behaviour change approach such as COMBI would be more appropriate. This method uses behavioural models, as well as communication and marketing theory and practice to instil change in practices. As Sokrin and Manderson stated, for any intervention to be successful, community involvement is crucial [50].

Awareness of dengue symptoms was found to be lower. To improve this, any dengue prevention program should also include education regarding dengue symptoms. Indeed, early testing and diagnosis is crucial to improving dengue outcomes, and mortality rates can be reduced when people are able to correctly identify dengue symptoms [51]. If improvements with regards to dengue prevention practices as well as symptom knowledge can be achieved, it could significantly improve dengue health outcomes in Cambodia.

### Disclaimer

The views expressed are those of the authors and do not necessarily represent the official policy or position of the Department of the Navy, Department of Defense, or the U.S. Government.

## Supporting information

S1 Checklist STROBE checklist. (DOCX)

S1 Survey Knowledge, attitudes and practices survey forms. (PDF)

S2 Survey Entomology survey forms. (PDF)

## References

[1] Brady OJ, Gething PW, Bhatt S, Messina JP, Brownstein JS, Hoen AG, et al Refining the Global Spatial Limits of Dengue Virus Transmission by Evidence-Based Consensus. PLoS Negl Trop Dis. 2012;6 doi: 10.1371/journal.pntd.0001760 22880140 10.1371/journal.pntd.0001760 PMC3413714

[2] World Health Organization. Dengue: guidelines for diagnosis, treatment, prevention, and control [Internet]. Special Programme for Research and Training in Tropical Diseases. 2009. doi:WHO/HTM/NTD/DEN/2009.1

[3] Bhatt S, Gething PW, Brady OJ, Messina JP, Farlow AW, Moyes CL, et al The global distribution and burden of dengue. Nature. Nature Publishing Group; 2013;496: 504–507. doi: 10.1038/nature12060 23563266 10.1038/nature12060 PMC3651993

[4] Capeding MR, Tran NH, Hadinegoro SRS, Ismail HIHM, Chotpitayasunondh T, Chua MN, et al Clinical efficacy and safety of a novel tetravalent dengue vaccine in healthy children in Asia: a phase 3, randomised, observer-masked, placebo-controlled trial. Lancet. 2014;384: 1358–1365. doi: 10.1016/S0140-6736(14)61060-6 25018116 10.1016/S0140-6736(14)61060-6

[5] Villar L, Dayan GH, Arredondo-García JL, Rivera DM, Cunha R, Deseda C, et al Efficacy of a Tetravalent Dengue Vaccine in Children in Latin America. N Engl J Med. Massachusetts Medical Society; 2015;372: 113–123. doi: 10.1056/NEJMoa1411037 25365753 10.1056/NEJMoa1411037

[6] Hahn H, Chastel C. Dengue in Cambodia in 1963. Nineteen laboratory-proved cases. Am J Trop Med Hyg. 1970; 10.4269/ajtmh.1970.19.106 5416281

[7] Shepard DS, Undurraga EA, Halasa YA. Economic and Disease Burden of Dengue in Southeast Asia. PLoS Negl Trop Dis. 2013;7 doi: 10.1371/journal.pntd.0002055 23437406 10.1371/journal.pntd.0002055 PMC3578748

[8] Khun S, Manderson LH. Abate distribution and dengue control in rural Cambodia. Acta Trop. 2007;101: 139–146. doi: 10.1016/j.actatropica.2007.01.002 17291439 10.1016/j.actatropica.2007.01.002

[9] World Health Organization. Global Strategy for Dengue Prevention and Control 2012–2020 [Internet]. World Health Organiszation 2012. doi:/entity/denguecontrol/9789241504034/en/index.html

[10] Ponlawat A, Scott JG, Harrington LC. Insecticide susceptibility of Aedes aegypti and Aedes albopictus across Thailand. J Med Entomol. 2005;42: 821–5. doi: 10.1603/0022-2585(2005)042[0821:ISOAAA]2.0.CO;2 16363166 10.1603/0022-2585(2005)042[0821:ISOAAA]2.0.CO;2

[11] Mulyatno KC, Yamanaka A, Ngadino, Konishi E. Resistance of Aedes aegypti (L.) larvae to temephos in Surabaya, Indonesia. Southeast Asian J Trop Med Public Health. 2012;43: 29–33. 23082551

[12] Chen CD, Nazni WA, Lee HL, Norma-Rashid Y, Lardizabal ML, Sofian-Azirun M. Temephos resistance in field Aedes (Stegomyia) albopictus (Skuse) from Selangor, Malaysia. Trop Biomed. 2013;30: 220–30. 23959487

[13] Polson KA, Curtis C, Seng CM, Olson JG, Chantha N, C S, et al Susceptibility of Two Cambodian Populations of Aedes aegypti Mosquito Larvae to Temephos During 2001. Dengue Bull–. 2001;25 Available: http://apps.who.int/iris/bitstream/10665/163688/1/dbv25p79.pdf

[14] Erlanger TE, Keiser J, Utzinger J. Effect of dengue vector control interventions on entomological parameters in developing countries: A systematic review and meta-analysis. Med Vet Entomol. Blackwell Publishing Ltd; 2008;22: 203–221. doi: 10.1111/j.1365-2915.2008.00740.x 18816269 10.1111/j.1365-2915.2008.00740.x

[15] Arunachalam N, Tana S, Espino F, Kittayapong P, Abeyewickreme W, Wai KT, et al Eco-bio-social determinants of dengue vector breeding: a multicountry study in urban and periurban Asia. Bull World Health Organ. 2010;88: 173–184. doi: 10.2471/BLT.09.067892 20428384 10.2471/BLT.09.067892 PMC2828788

[16] Castro M, Sánchez L, Pérez D, Carbonell N, Lefèvre P, Vanlerberghe V, et al A community empowerment strategy embedded in a routine dengue vector control programme: a cluster randomised controlled trial. Trans R Soc Trop Med Hyg. 2012;106: 315–321. doi: 10.1016/j.trstmh.2012.01.013 22465423 10.1016/j.trstmh.2012.01.013

[17] Andersson N, Nava-Aguilera E, Arosteguí J, Morales-Perez A, Suazo-Laguna H, Legorreta-Soberanis J, et al Evidence based community mobilization for dengue prevention in Nicaragua and Mexico (Camino Verde, the Green Way): cluster randomized controlled trial. BMJ. 2015;351: h3267 doi: 10.1136/bmj.h3267 26156323 10.1136/bmj.h3267 PMC4495677

[18] Beauté J, Vong S. Cost and disease burden of dengue in Cambodia. BMC Public Health. 2010;10: 521 doi: 10.1186/1471-2458-10-521 20807395 10.1186/1471-2458-10-521 PMC2941493

[19] National Institute of Statistics, Directorate General for Health, ICF International. Cambodia Demographic and Health Survey [Internet]. 2015. Available: http://dhsprogram.com/pubs/pdf/FR312/FR312.pdf

[20] Hustedt J, Doum D, Keo V, Ly S, Sam B, Chan V, et al Determining the efficacy of guppies and pyriproxyfen (Sumilarv® 2MR) combined with community engagement on dengue vectors in Cambodia: study protocol for a randomized controlled trial. Trials. 2017;18 doi: 10.1186/s13063-017-2105-2 28778174 10.1186/s13063-017-2105-2 PMC5545006

[21] The DHS Program. DHS Model Questionnaire—Phase 7 (English) [Internet]. 2015 [cited 26 Jul 2016]. Available: http://dhsprogram.com/publications/publication-dhsq7-dhs-questionnaires-and-manuals.cfm

[22] Knox TB, Yen NT, Nam VS, Gatton ML, Kay BH, Ryan PA. Critical evaluation of quantitative sampling methods for Aedes aegypti (Diptera: Culicidae) immatures in water storage containers in Vietnam. J Med Entomol. The Oxford University Press; 2007;44: 192–204. doi: 10.1093/jmedent/44.2.192 17427686 10.1603/0022-2585(2007)44[192:ceoqsm]2.0.co;2

[23] Filmer D, Pritchett LH. Estimating Wealth Effects Without Expenditure Data—or Tears: Demography. 2001;38: 115–132. doi: 10.1353/dem.2001.0003 11227840 10.1353/dem.2001.0003

[24] McKenzie D. Measuring Inequality with Aset Indicators. BREAD Work Pap No 042. 2004;

[25] Swaddiwudhipong W, Lerdlukanavonge P, Khumklam P, Koonchote S, Nguntra P, Chaovakiratipong C. A survey of knowledge, attitude and practice of the prevention of dengue hemorrhagic fever in an urban community of Thailand. Southeast Asian J Trop Med Public Heal. 1992;23: 207–211. 1439972

[26] Koenraadt CJM, Tuiten W, Sithiprasasna R, Kijchalao U, Jones JW, Scott TW. Dengue knowledge and practices and their impact on Aedes aegypti populations in Kamphaeng Phet, Thailand. Am J Trop Med Hyg. 2006;74: 692–700. doi:74/4/692 [pii] 16607007

[27] Tsuzuki A, Huynh T, Tsunoda T, Luu L, Kawada H, Takagi M. Effect of existing practices on reducing Aedes aegypti pre-adults in key breeding containers in ho Chi Minh city, vietnam. Am J Trop Med Hyg. 2009;80: 752–757. 19407119

[28] Mayxay M, Cui W, Thammavong S, Khensakhou K, Vongxay V, Inthasoum L, et al Dengue in peri-urban Pak-Ngum district, Vientiane capital of Laos: a community survey on knowledge, attitudes and practices. BMC Public Health. 2013;13: 434 doi: 10.1186/1471-2458-13-434 23641953 10.1186/1471-2458-13-434 PMC3645963

[29] Paz-Soldán VA, Morrison AC, Lopez JJC, Lenhart A, Scott TW, Elder JP, et al Dengue knowledge and preventive practices in Iquitos, Peru. Am J Trop Med Hyg. 2015;93: 1330–1337. doi: 10.4269/ajtmh.15-0096 26503276 10.4269/ajtmh.15-0096 PMC4674254

[30] Maude RJ, Nguon C, Ly P, Bunkea T, Ngor P, Canavati de la Torre SE, et al Spatial and temporal epidemiology of clinical malaria in Cambodia 2004–2013. Malar J. BioMed Central; 2014;13: 385 doi: 10.1186/1475-2875-13-385 25266007 10.1186/1475-2875-13-385 PMC4531392

[31] World Health Organization, UNICEF. Cambodia: estimates on the use of water sources and sanitation facilities (1980–2015) [Internet]. 2015 [cited 15 Aug 2016]. Available: http://www.wssinfo.org/documents/?tx_displaycontroller%5Btype%5D=country_files&tx_displaycontroller%5Bsearch_word%5D=Cambodia

[32] Seng CM, Setha T, Nealon J, Chantha N, Socheat D, Nathan MB. The effect of long-lasting insecticidal water container covers on field populations of Aedes aegypti (L.) mosquitoes in Cambodia. J Vector Ecol. 2008;33: 333–41. doi: 10.3376/1081-1710-33.2.333 19263854 10.3376/1081-1710-33.2.333

[33] Lan NT, Cam LT, Chuong NP, Tri L, Fonsmark L, Poulsen A, et al The Impact of Health Education on Mother ‘ s Knowledge, Attitude and Practice (KAP) of Dengue Haemorrhagic Fever. Dengue Bull. 27: 174–180.

[34] Malhotra V, Kaur P. The Community knowledge, attitude and practices regarding Dengue fever in field practice area of urban training health centre of Patiala. 2014;2: 19–26.

[35] Dégallier N, Vilarinhos PT, de Carvalho MS, Knox MB, Caetano J. People’s knowledge and practice about dengue, its vectors, and control means in Brasilia (DF), Brazil: its relevance with entomological factors. J Am Mosq Control Assoc. 2000;16: 114–123. 10901634

[36] Hairi F, Ong C-HS, Suhaimi A, Tsung T-W, bin Anis Ahmad MA, Sundaraj C, et al A knowledge, attitude and practices (KAP) study on dengue among selected rural communities in the Kuala Kangsar district. Asia Pac J Public Health. 2003;15: 37–43. doi: 10.1177/101053950301500107 14620496 10.1177/101053950301500107

[37] Shuaib F, Todd D, Campbell-Stennett D, Ehiri J, Jolly PE. Knowledge, attitudes and practices regarding dengue infection in Westmoreland, Jamaica. West Indian Med J. 2010;59: 139–146. doi: 10.1055/s-0029-1237430.Imprinting 21132094 PMC2996104

[38] World Health Organization, Cambodia M of H. Health Service Delivery Profile Cambodia 2012 Compiled in collaboration between WHO and Ministry of Health, Cambodia Cambodia health service delivery profile [Internet]. 2012. Available: http://www.wpro.who.int/health_services/service_delivery_profile_cambodia.pdf

[39] Anders KL, Nguyet NM, Chau NVV, Hung NT, Thuy TT, Lien LB, et al Epidemiological factors associated with dengue shock syndrome and mortality in hospitalized dengue patients in Ho Chi Minh City, Vietnam. Am J Trop Med Hyg. 2011;84: 127–134. doi: 10.4269/ajtmh.2011.10-0476 21212214 10.4269/ajtmh.2011.10-0476 PMC3005500

[40] Elsinga J, Lizarazo EF, Vincenti MF, Schmidt M, Velasco-Salas ZI, Arias L, et al Health Seeking Behaviour and Treatment Intentions of Dengue and Fever: A Household Survey of Children and Adults in Venezuela. Matovu E, editor. PLoS Negl Trop Dis. Public Library of Science; 2015;9: e0004237 doi: 10.1371/journal.pntd.0004237 26624283 10.1371/journal.pntd.0004237 PMC4666462

[41] Khun S, Manderson L. Health seeking and access to care for children with suspected dengue in Cambodia: An ethnographic study. BMC Public Health. BioMed Central; 2007;7: 262 doi: 10.1186/1471-2458-7-262 17892564 10.1186/1471-2458-7-262 PMC2164964

[42] Matta S, Bhalla S, Singh D, Rasania SK, Singh S. Knowledge, Attitude & Practice (KAP) on Dengue fever: A Hospital Based Study Book Review. Indian J community Med. 2006;31: 2–3.

[43] Muny M. Survey Report on Waste Management Practices At Municipality/District Level [Internet]. 2016. Available: http://www.delgosea.eu/cms/News/Results-from-Waste-Management-Survey-in-Cambodia-Published

[44] Oria D, Acosta S, Ana D, Chalgub M, Osvaldo R, González B. Modificación de los conocimientos, actitudes y prácticas de la población sobre la prevención de los mosquitos. Rev Cubana Hig Epidemiol. 1999;37: 6–12.

[45] Ana Margarita de la Cruz L, Denys Figueroa L, Leonardo Chacón L, Maritza Gómez L, Díaz M, Finlay CM. Conocimientos, opiniones y prácticas sobre Aedes aegypti. Rev Cubana Med Trop. 1999;51: 135–7. 10887576

[46] Huy R, Wichmann O, Beatty M, Ngan C, Duong S, Margolis HS, et al Cost of dengue and other febrile illnesses to households in rural Cambodia: a prospective community-based case-control study. BMC Public Health. BioMed Central; 2009;9: 155 doi: 10.1186/1471-2458-9-155 19473500 10.1186/1471-2458-9-155 PMC2696434

[47] Damme W Van, Leemput L Van, Por I, Hardeman W, Meessen B, Ouma J, et al Out-of-pocket health expenditure and debt in poor households: Evidence from Cambodia. Tropical Medicine and International Health. BioMed Central; 2004 pp. 273–280. doi: 10.1046/j.1365-3156.2003.01194.x 15040566 10.1046/j.1365-3156.2003.01194.x

[48] Wichmann O, Yoon I-K, Vong S, Limkittikul K, Gibbons R V., Mammen MP, et al Dengue in Thailand and Cambodia: An Assessment of the Degree of Underrecognized Disease Burden Based on Reported Cases. Guzman MG, editor. PLoS Negl Trop Dis. World Health Organization; 2011;5: e996 doi: 10.1371/journal.pntd.0000996 21468308 10.1371/journal.pntd.0000996 PMC3066139

[49] Huy R, Buchy P, Conan A, Ngan C, Ong S, Ali R, et al National dengue surveillance in Cambodia 1980–2008: epidemiological and virological trends and the impact of vector control. Bull World Health Organ. 2010;88: 650–657. doi: 10.2471/BLT.09.073908 20865069 10.2471/BLT.09.073908 PMC2930366

[50] Khun S, Manderson L. Community and school-based health education for dengue control in rural Cambodia: A process evaluation. Lennon J, editor. PLoS Negl Trop Dis. Ministry of Health; 2007;1: e143 doi: 10.1371/journal.pntd.0000143 18160981 10.1371/journal.pntd.0000143 PMC2154392

[51] Kittigul L, Suankeow K, Sujirarat D, Yoksan S. Dengue hemorrhagic fever: Knowledge, attitude and practice in Ang Thong Province, Thailand. Southeast Asian J Trop Med Public Health. 2003;34: 385–392. 12971568

