# Supplementary material for: Dengue knowledge, attitudes and practices and their impact on community-based vector control in rural Cambodia
Source: PLoS Negl Trop Dis. 2018 Feb 16;12(2):e0006268. doi: 10.1371/journal.pntd.0006268 (PMC5833285; doi:10.1371/journal.pntd.0006268)
Supplement: S1 Survey — (PDF) [file pntd.0006268.s002.pdf]

កម្រងសំនួរសម្រាប់ខ្នងផ្ទះ

Questionnaire_HH_Final

អត្តសញ្ញាណកម្ម IDENTIFICATION
លេខកូដសិក្សាខ្នងផ្ទះ |____|____|____|____|
Household Study ID Number

**Q1. លេខទូរស័ព្ទ (ខ្នងផ្ទះ)**<br>Tel: (+855)________________	**Q4. GPS (location in GPS name)** ________<br>X: _________________________<br>Y: _________________________
**Q2. ខេត្ត** |___| Province name/code: _________________	**Q5. ការិយាល័យសុខាភិបាលស្រុកប្រតិបត្ត** |___|___| OD name/code:_____________________
**Q3. ភូមិ** |__|__|__|__| Village name/code:	**Q6. ឈ្មោះអ្នកសំភាសន៍** |___|___| Name of Interviewer/code:

សេចក្តីណែនាំ: ជម្រាបសួរ ខ្ញុំបាទ នាងខ្ញុំឈ្មោះ__________________ខ្ញុំធ្វើការជាមួយក្រសួងសុខាភិបាល និងអង្គការម៉ាឡារាខុនសតធៀម ដើម្បីមករៀនសូត្រ និងស្វែងយល់ស្តីពីវិធីនៃការការពារជំងឺគ្រុនឈាម។ យើងខ្ញុំចង់រៀនពី ការយល់ដឹង ឥរិយាបទ និង វិធីការពារនានាដែលអ្នកបានអនុវត្តន៍នៅផ្ទះ។ ដើម្បីសិក្សារៀនសូត្រពីវិធីនេះ យើងខ្ញុំសូមសួរនូវសំណួរ មួយចំនួនដូចខាងក្រោម។ យើងខ្ញុំនឹងធ្វើការអង្កេតពីទីកន្លែងបង្កកំណើតរបស់មូស និងវិធីសាស្រ្តនៃការការពារនៅក្នុង និងក្រៅផ្ទះរបស់អ្នក។ យើងខ្ញុំនឹងសួរចំនួន២០នាទី។ ការចូលរួមឆ្លើយសំនួររបស់អ្នកគឺរក្សាដោយសំងាត់ និង ដោយ ស្ម័គ្រចិត្ត។ បើអ្នកយល់ព្រមឆ្លើយសំនួរ អ្នកក៏អាចបដិសេធ ឬក៏ឈប់ឆ្លើយសំនួរក៏បាន។ រាល់ចំលើយរបស់អ្នកនឹង ជួយដល់យើងខ្ញុំក្នុងការអភិវឌ្ឍន៍នូវការបង្ការ និងទំនួលខុសត្រូវចំពោះជំងឺគ្រុនឈាមក្នុងប្រទេសកម្ពុជា។ ជាងនេះទៅទៀត នឹងជួយដល់ការបង្កើតនូវចំណេះដឹង ដែលអ្នកនឹងរៀនពីវិធីដ៏មានតម្លៃដើម្បីការពារជំងឺគ្រុនឈាម និងកត្តាជំងឺចម្លង នានានៅក្នុងផ្ទះ និងសហគមន៍របស់អ្នក។ សូមគិតថានេះមិនមែនជាការស្វែងរកចំឡើយខុសត្រូវឡើយ។ តើអ្នកមានសំនួរអ្វីទេ? បើគ្មានសំនួរ សូមអនុញ្ញាតិឲ្យសួរ?

ហត្ថលេខា/ឈ្មោះ (Signature/Name)**:** ________________ ថ្ងៃទីខែឆ្នាំ (Date)**:** ________ (DD/MM/YYYY)

អ្នកបញ្ចូលទិន្នន័យ (Data entry) 1st : _________________ ថ្ងៃទីខែឆ្នាំ (Date): ________ (DD/MM/YYYY)

2nd: _________________ ថ្ងៃទីខែឆ្នាំ (Date): ________ (DD/MM/YYYY)

ផ្នែកទី ១ ស្ថានភាពសេដ្ឋកិច្ចប្រជាសាស្ត្រ Section 1: Socio-demographics

លរ/រ.<br>No.	សំណួរ<br>QUESTION	ប្រភេទលេខកូដ<br>CODING CATEGORIES		រំលង<br>Skip
Q101	**តើអ្នកមានអាយុប៉ុន្មាន?**<br>How old are you?	**ចំនួនឆ្នាំ**: |____|____| (Age)		
Q102	**ភេទអ្វី?**<br>What is your gender?	**ប្រុស**<br>(Male)	1	
		**ស្រី**<br>(Female)	2	
Q103	**តើអ្នកមានដើមកំណើតជាជនជាតិអ្វី?**<br>What ethnic group do you identify with?<br>**(ចម្លើយមានតែមួយ)**<br>**(Only 1 answers)**	**ខ្មែរ**<br>(Khmer)	1	
		**ចាម**<br>(Cham)	2	
		**វៀតណាម**<br>(Vietnamese)	3	
		**ផ្សេងៗ បញ្ជាក់** ________________<br>Other (specify)	98	
Q104	**តើអ្នកទទួលបានការអប់រំខ្ពស់បំផុតកម្រិតណា?**<br>What was the highest level of school attended by you/household head completed?<br>**(ចម្លើយមានតែមួយ)**<br>**(Only 1 answers)**	**មិនដែលបានរៀន**<br>(None)	0	
		**សាលាក្រៅផ្លូវការ (រៀននៅវត្ត.....)**<br>(Unofficial school)	1	
		**បឋមសិក្សា (1-6)**<br>(Primary school, 1-6)	2	
		**អនុវិទ្យាល័យ (7-9)**<br>(Secondary school, 7-9)	3	
		**វិទ្យាល័យ (10-12)**<br>(High school, 10-12)	4	
		**ថ្នាក់មហាវិទ្យាល័យ**<br>(University level)	5	
Q105	**តើមុខរបរអ្វីដែលជាចំណូលចម្បងសម្រាប់ទំនុក បម្រុងក្នុងគ្រួសារ?**<br>What is your main occupation?<br>**(ចម្លើយមានតែមួយ)**<br>**(Only 1 answers)**	**គ្មានមុខរបរ ឬ នៅផ្ទះ**<br>None/Stay at home	0	
		**កសិករ**<br>Farmer	1	
		**បុគ្គលិកមន្ត្រីរាជការស៊ីវិល**<br>Civil government staff	2	
		**បុគ្គលិកមន្ត្រីរាជការ ប្រដាប់អាវុធ (ប៉ូលីស/ទាហាន...)**<br>Security government staff	3	
		**បុគ្គលិកក្រុមហ៊ុនឯកជន**<br>Company staff	4	

លរ/រ. No.	សំណួរ QUESTION	ប្រភេទលេខកូដ CODING CATEGORIES			រំលង Skip
		បុគ្គលិកមន្ត្រីក្រៅរដ្ឋាភិបាល NGO Staff		5	
		កម្មករលក់កម្លាំងពលកម្ម Labor worker		6	
		អ្នកលក់ដូរតាមផ្ទះ ឬផ្សារ Sell vender or market seller		7	
		ផ្សេងៗ_______________ Other		98	
Q106	តើក្នុងផ្ទះរបស់អ្នកមានសម្ភារៈប្រើប្រាស់ទាំងអស់នេះដែរឬទេ?<br><br>Does your household have<br><br>**ចម្លើយអាចមានច្រើន**<br>**Multiple Answers**		**Yes**	**No**	
		អគ្គិសនី (បណ្តាញអគ្គិសនី រដ្ឋ ឬឯកជន) Electricity (electricity cable state or private)	1	0	
		វិទ្យុ Radio	1	0	
		ទូរទស្សន៍ TV	1	0	
		ទូរស័ព្ទ Mobile Phone	1	0	
		ទូរទឹកកក Refrigerator	1	0	
		ទូញួ ខោអាវ A Wardrobe	1	0	
		ម៉ាស៊ីនដេរ A sewing machine or loom	1	0	
		ក្បាលចាក់ឌីស A CD/DVD/MP3 player	1	0	
		ម៉ាស៊ីនភ្លើង អាគុយ ឬ បន្ទះប្រើពន្លឺព្រះអាទិត្យ Generator/ Battery/Solar power	1	0	

លរ/រ. No.	សំណួរ QUESTION	ប្រភេទលេខកូដ CODING CATEGORIES			រំលង Skip
		ផ្សេងៗ ____________________ Other	1	0	

**Q107**	**តើមានសម្ភារៈអ្វីខ្លះ នៅក្នុងផ្ទះរបស់អ្នក ដូចខាងក្រោម?**<br><br>Does any member of this household own:<br><br>**ចម្លើយអាចមានច្រើន**<br>**Multiple Answers**		**Yes**	**No**	
		**នាឡិការដៃ**<br>Hand Watch	1	0	
		**កង់**<br>Bicycle/cyclo	1	0	
		**ម៉ូតូ**<br>Motorcycle/scooter	1	0	
		**ម៉ូតូកង់បី**<br>Motorcycle-cart	1	0	
		**គោ ក្របី**<br>Oxcart/Horse cart	1	0	
		**ឡាន**<br>Car/Truck/Van	1	0	
		**ទូកមានម៉ាស៊ីន**<br>Boat with motor	1	0	
		**ទូក**<br>Boat without motor	1	0	
		**ផ្សេងៗ** ____________________<br>Other	1	0	
**Q108**	**តើអ្នកប្រើចំហេះអ្វីខ្លះ សម្រាប់ចំអិនម្ហូបអាហារ នៅក្នុងផ្ទះរបស់អ្នក**<br><br>What type of fuel does your household use for cooking?<br><br>**ចម្លើយអាចមានច្រើន**<br>**Multiple Answers**		Yes	No	
		**អគ្គិសនី**<br>Electricity	1	0	
		**ចង្ក្រានហ្គាស**<br>LPG	1	0	
		**ឡូឧស្ម័ន/ជីវៈឧស្ម័ន**<br>Biogas	1	0	
		**ធ្យូង**<br>Charcoal	1	0	
		**ឱ្យស**<br>Wood	1	0	

		**ចម្បើង ឬ ស្លឹករុក្ខជាតិ**<br>Straw/shrubs/grass	1	0	
		**គ្រាប់ធញ្ញជាតិ (ឧ. ស្នូលពោត....)**<br>Agriculture crop	1	0	
		**លាមក សត្វ**<br>Animal Dung	1	0	
		**គ្មានអាហារចំអិនក្នុងផ្ទះ**<br>No food cooked in household	1	0	
		**ផ្សេងៗ**_________________	1	0	

□□□□□□□□ □ □□□□□□□□□□□□□□□□□□□□□□□□□□□□□□□ **Section 2: Knowledge about dengue**

លរ/ស. No.	សំណួរ QUESTION	ប្រភេទលេខកូដ CODING CATEGORIES			រំលង Refuse
**Q201**	**តើជំងឺគ្រុនឈាម ឆ្លងដោយសារអ្វី?**<br>How is dengue transmitted?	**មូសខាំ**<br>Mosquito bite	1		
		**ផ្សេងទៀត បញ្ជាក់**______________<br>Other (Specify)	98		
		**មិនដឹង**<br>Don't know	99		→Q204
**Q202**	**តើភាគច្រើនមូសបង្កជំងឺគ្រុនឈាម (មូសខ្លា) ខាំនៅពេលណា?**<br>When do dengue mosquitos **most often** bite?<br>**(ចម្លើយមានតែមួយ)**<br>**(Only 1 answers)**	**មូសខាំ នៅពេលថ្ងៃ**<br>Bite during the day	1		
		**មូសខាំ នៅពេលយប់**<br>Bite during the night time	2		
		**មិនដឹង**<br>Don't know	99		
**Q203**	**តើមូសអាចបង្កើតកូននៅកន្លែងណាខ្លះ?**<br>Where can mosquitos breed?<br>**ចម្លើយអាចមានច្រើន**<br>**Multiple Answers**		**Yes**	**No**	
		**មិនដឹង**<br>Don't know	1	0	
		**ក្នុងទឹកពាង**<br>Water storage jars	1	0	
		**ក្នុងទឹក ដក់ក្នុងសំបកដូង កំប៉ុង**<br>Coconut shells /Cans	1	0	
		**ក្នុងអាងងូតទឹក (**Cement baths)	1	0	
		**ទឹកដក់ក្នុងសំបុកត្រោស ឬ ប្រហោងតូចៗលើដើមឈើជុំវិញផ្ទះ**<br>Ant traps	1	0	
		**ទឹកដក់ក្នុងកន្លែងដែលអាចដក់ទឹកបាននៅជុំវិញផ្ទះរបស់អ្នក**<br>Anything with water around your environment	1	0	
		**ក្នុងសំបកកង់ឡាន ម៉ូតូ** (Tires)	1	0	
		**ផ្សេងទៀត បញ្ជាក់** ____________	1	0	
**Q204**	**តើអ្នកធ្វើដូចម្តេចខ្លះ ដើម្បីការពារមូសមិនអោយបង្កើតកូនតទៅទៀតបាន?**<br>How can you prevent mosquitos from breeding?<br>***អាចមានចំលើយច្រើន***<br>***ចូរគូសរង្វង់ជុំវិញចំលើយទាំងអស់***<br>***ចូរសួរដេញរកតើមានអ្វីផ្សេងទៀតឬអត់?***		**Yes**	**No**	
		**មិនដឹង**<br>Don't know	1	0	
		**ដាក់ថ្នាំអាបែត**<br>Use Abate	1	0	
		**ប្រើ PPF**<br>Use PPF	1	0	

ល/រ.<br>No.	សំណួរ<br>QUESTION	ប្រភេទលេខកូដ<br>CODING CATEGORIES			រំលង<br>Refuse
	MULTIPLE RESPONSES POSSIBLE<br>CIRCLE ALL MENTIONED<br>PROBE ONCE: ANYTHING ELSE?	**ប្តូរទឹកក្នុងពាងញឹកញាប់**<br>Changing stored water frequently	1	0	
		**ផ្កាប់ធុងចាស់ៗចុះក្រោម**<br>Turn containers upside down	1	0	
		**ប្រើគំរបពាង**<br>Put lids on water jars	1	0	
		**ដាក់ត្រីក្នុងពាង**<br>Put fish in water jars	1	0	
		**បាញ់ស្រ្តាយ (ថ្នាំមូស)**<br>Spraying insecticide	1	0	
		**ផ្សេងទៀត**_______________<br>Other (Specify)	1	0	
**Q205**	**ដើម្បីកុំអោយមូសខាំ តើអ្នកការពារខ្លួន ឬក៏សមាជិកអ្នកគ្រួសាររបស់អ្នក ដោយរបៀបណា?**<br><br>How can you prevent mosquitos from biting you or your family?<br><br>***អាចមានចំលើយច្រើន***<br>***ចូរគូសរង្វង់ជុំវិញចំលើយទាំងអស់***<br>***ចូរសួរដេញរកតើមានអ្វីផ្សេងទៀតឬអត់?***<br><br>MULTIPLE RESPONSES POSSIBLE<br>CIRCLE ALL MENTIONED<br>PROBE ONCE: ANYTHING ELSE?		Yes	No	
		**មិនដឹង**<br>Don’t know	1	0	
		**ដុត ឬ កប់ សំបកដូង**<br>Burn/Bury Coconut Shells	1	0	
		**ស្លៀកពាក់ខោអាវវែងៗ**<br>Wear long sleeves/long pants	1	0	
		**លាបថ្នាំកំចាត់មូស**<br>Use mosquito repellent	1	0	
		**ដេកក្នុងមុងពេលដេកថ្ងៃ**<br>Use mosquito net during day	1	0	
		**កាប់ស្មៅគុម្ពោតស៊ប់ទ្រុបនៅជិតផ្ទះ**<br>Cut down bushes near the house	1	0	
		**ឲ្យក្មេងៗលេងឆ្ងាយពីជម្រកមូស**<br>Have children play far from mosquito breeding areas	1	0	

លរ/រ. No.	សំណួរ QUESTION	ប្រភេទលេខកូដ CODING CATEGORIES			រំលង Refuse
		ដុតធូបមូសពេលថ្ងៃ<br>Use mosquito coils during the day	1	0	
		សំអាតជុំវិញបរិវេណផ្ទះ<br>Keep household environment clean	1	0	
		បត់ខោអាវឲ្យមានរបៀប<br>Keep cloths tidy	1	0	
		ប្រដាប់ឆក់មូស<br>Electricity trap	1	0	
		ប្រើកង្ហារដេញមូស<br>Use fan	1	0	
		ផ្សេង________________	1	0	
**Q206**	**តើរោគសញ្ញាជំងឺគ្រុនឈាមមានអ្វីខ្លះ?**		Yes	No	
	What are the symptoms of dengue?	**មិនដឹង (**Don't know)	1	0	
	អាចមានចំលើយច្រើន	**គ្រុនក្តៅ (**Fever)	1	0	
	ចូរគូសរង្វង់ជុំវិញចំលើយទាំងអស់	**ឈឺក្បាល (**Headache)	1	0	
	ចូរសួរដេញរកតើមានអ្វីផ្សេងទៀតឬអត់?	**សន្លឹម (**Somnolence)	1	0	
	**MULTIPLE RESPONSES POSSIBLE**	**ក្អួត ចង្អោរ (**Nausea/vomiting)	1	0	
	**CIRCLE ALL MENTIONED**	**កន្ទួលក្រហមៗ (**Rash)	1	0	
	**PROBE ONCE: ANYTHING ELSE?**	**ឈឺឆ្អឹងឆ្អែង និងចុកចាប់**<br>Aches and Pains/Body pain	1	0	
		**ធ្លាក់ឈាម (**Bleeding)	1	0	
		**អាការៈស្ទើរសន្លប់**<br>Shock	1	0	
		**ឈឺសាច់ដុំ**<br>Muscular Pain	1	0	
		**ផ្សេងៗ** _______________<br>Other (Specify)	1	0	
**Q207**	**តើអ្នកដឹងពី ពេលដែលជម្ងឺ គ្រុនឈាមកើតឡើង ដែរ ឬ ទេ នៅក្នុងពេលមួយឆ្នាំៗ?**<br>Are there certain times a year when you recognize that more people in your	**ទេ**<br>No		0	**→Q301**
		**បាទ**<br>Yes		1	

ល/រ. No.	សំណួរ QUESTION	ប្រភេទលេខកូដ CODING CATEGORIES		រំលង Refuse
	family /village get sick of dengue?	**មិនដឹង** Don’t know	99	
**Q208**	**តើជម្ងឺគ្រុនឈាមកើតឡើងនៅពេលណា? (ខែណា ដល់ខែណា)** If yes, when?	**ចាប់ពីខែ: _______ ដល់ខែ: _______** **From: ..... to .....**		
		**មិនដឹង** Don’t know	99	

□□□ **(Section 3: Health Seeking Behaviour)**

ល/រ. No.	សំណួរ QUESTION:	ប្រភេទលេខកូដ CODING CATEGORIES			រំលង Refuse
**Q301**	**បើមានសមាជិកគ្រួសាររបស់អ្នកគ្រុនក្តៅ តើអ្នកនឹងធ្វើអ្វីមុនគេបង្អស់?**<br><br>If you think you or someone in your family has fever, what would you do **First?**<br><br>**(ចម្លើយមានតែមួយគត់)**<br>**(Only 1 answer)**	**សេវាសុខភាពសាធារណៈ(រដ្ឋ)**<br>Go to Health Facility		1	
		**ពេទ្យឯកជន (**Go to Private Provider)		2	
		**អ្នកស្ម័គ្រចិត្តសុខភាពភូមិ**<br>Go to Community Health Worker		3	
		**ទិញថ្នាំនៅហ្វាម៉ាស៊ី** (Take Drugs From Pharmacy)		4	
		**សំរាកនៅផ្ទះ ឬ រងចាំរហូតបាត់ គ្រុនក្តៅដោយខ្លួនឯង**<br>Stay at home/wait for fever to go away		5	
		**ផ្សេងៗ** _______________		6	
		**មិនដឹង (**Don't know)		99	
**Q302**	**ប្រសិនបើមានសមាជិកគ្រួសារណាម្នាក់មានរោគ សញ្ញាគ្រុនក្តៅ តើរយៈប៉ុន្មានថ្ងៃទើបអ្នកទៅរកការព្យាបាល?**<br>If your family member gets fever, how many days do you wait to seek care after symptoms start?	|___|___| **កត្រាលេខ 0 ប្រើសិនបើក្នុងថ្ងៃគ្រុនក្តៅ**<br>**កត្រាលេខ ៩៩ ប្រសិនបើមិនដឹង**<br>Record **"0"** if they respond in the same day<br>Record **"99"** if they respond Don't Know			
**Q303**	**បើសិនជាអ្នកសង្ស័យថាមានណាម្នាក់កើតជម្ងឺគ្រុនឈាម តើកន្លែងណាដែលអ្នកនឹងណែនាំ ឬក៏ទៅធ្វើរោគវិនិច្ឆ័យ?**<br><br>If you suspect someone in your family has dengue, where would you go for advice/testing?<br><br>**(ចម្លើយមានច្រើន)**<br>**(Multiple answers)**		**Yes**	**No**	
		**សេវាសុខភាពសាធារណៈ(រដ្ឋ)**<br>Go to Health Facility	1	0	
		**ពេទ្យឯកជន**<br>Go to Private Provider	1	0	
		**ទៅរកអ្នកស្ម័គ្រចិត្តសុខភាពភូមិ**<br>Go to Community Health Worker	1	0	
		**ទិញថ្នាំនៅហាងលក់ថ្នាំ (**Pharmacy)	1	0	
		**ទៅជួបអ្នកដឹកនាំសាសនា (ព្រះសង្ឃ អាចារ្យ ឬ អ្នកផ្លុំ ស្តោះជាដើម)**<br>Religious Leader	1	0	
		**ផ្សេង** _______________	1	0	

□□□□□□□ □ □□□□□□□ **(Section 4: Attitude)**

ល/រ. No.	សំណួរ QUESTION:	ប្រភេទលេខកូដ CODING CATEGORIES		រំលង Refuse
**Q401**	**តើជំងឺគ្រុនឈាម ជាជំងឺធ្ងន់ធ្ងរមែនដែរ ឬ ទេ?**<br>Dengue is a serious illness?	**ទេ**<br>No	0	
		**បាទ**<br>Yes	1	

លរ. No.	សំណួរ QUESTION:	ប្រភេទលេខកូដ CODING CATEGORIES		បដិសេធ Refuse
		**មិនដឹង** Don't know	99	
**Q402**	**តើអ្នកពិតជាប្រឈមនឹងគ្រោះថ្នាក់ដែរ ឬ ទេ នៅពេលកើតជំងឺគ្រុនឈាម?**<br><br>You are at risk of getting dengue	**ទេ** No	0	
		**បាទ** Yes	1	
		**មិនដឹង** Don't know	99	
**Q403**	**តើជំងឺគ្រុនឈាមអាចបង្ការមុនបានដែរឬ ទេ?**<br><br>Dengue fever can be prevented	**ទេ** (No)	0	
		**បាទ** (Yes)	1	
		**មិនដឹង** (Don't know)	99	

□□□□□□□□ □ □□□□□□□□□□□□□□ (Section 5: Practices)

លរ/រ.<br>No.	សំណួរ<br>QUESTION:	ប្រភេទលេខកូដ<br>CODING CATEGORIES			រំលង<br>Skip
**Q501**	**តើអ្នកប្តូរទឹក (ពាង អាង....) ញឹកញាប់ប៉ុណ្ណា?**<br>How often do you change the storage water?<br>**(ចម្លើយមានតែមួយ)**<br>**(Only 1 answers)**	**មិនដែរប្តូរសោះ**<br>Not at all	0		
		**ច្រើនជាងមួយអាទិត្យម្តង**<br>More than once a week	1		
		**ម្តងក្នុងមួយអាទិត្យ**<br>Once time per week	2		
		**ពីរដងក្នុងមួយខែ**<br>Twice per month	3		
		**ច្រើនជាង ១ ខែម្តង**<br>More than 1 time per time	4		
		**ផ្សេងៗ** ______________________<br>Other (Specify)	5		
**Q502**	**តើអ្នកមានពាងទឹកធំៗ ចាប់ពី ៥០ លីត្រ ចំនួនប៉ុន្មាន? ហើយមានគម្រប ប៉ុន្មាន? ចំនួនពាងដាក់ត្រី?**<br>How many large water jars (>50L) do you have and how many are covered	**1.ចំនួនពាង** |_____|_____| Number of Jars			
		**2.ចំនួនគម្របពាង** |_____|_____| Number covered			
		**3. ចំនួនពាងដាក់ត្រី** |______|______| **សូមរំលងទៅសំនួរ Q504 ប្រសិនបើចម្លើយ ស្មើសូន្យ "00"**<br>Number of jars put fish<br>If record "00" go to Q504			
**Q503**	**បើសិនជាត្រីងាប់ តើអ្នកទៅយកនៅទីណា?**<br>If the fish die where would go to replace them?<br>**(ចម្លើយមានច្រើន)**<br>**(Multiple answers)**		Yes	No	
		**មិនដាក់ថែម**<br>Not Replace Them	1	0	
		**នៅមណ្ឌលសុខភាព**<br>Health Center	1	0	
		**នៅផ្ទះអ្នកស្ម័គ្រចិត្តសុខភាពភូមិ**<br>Community Health Worker	1	0	
		**ផ្សេង** ....<br>Other (specify)	1	0	
		**មិនដឹង**<br>Don't Know	1	0	
**Q504**	**តើអ្នកស្គាល់ PPF ដែរ ឬ ទេ?**<br>Do you know what PPF is?	**ទេ**<br>No	0		
		**បាទ/ចា**<br>Yes	1		
**Q505**	**តើអ្នកធ្វើដូចម្តេចខ្លះជាមួយសំបកដូង ឬ វត្ថុដែល អាច ដក់ទឹកបាន?**<br>What do you do with empty coconuts or containers?<br>**(ចម្លើយមានតែមួយ)**<br>**(Only 1 answers)**	**អត់ធ្វើអ្វីទាំងអស់**<br>Nothing	0		
		**ផ្កាប់ចោល**<br>Turn them upside down	1		
		**ដុត ឬ ច្រកទុក**<br>Burn them/put them in trash bag	2		
		**ផ្សេងៗ** ______________________<br>Other (Specify)	3		
**Q506**	**តើអ្នកចងមុងឬទេ នៅពេលសម្រាកពេលថ្ងៃ?**<br>How often do you use mosquito nets during daytime naps?	**ចំនួនថ្ងៃចងមុង ក្នុង 1 សប្តាហ៍ |____|**<br>day/week			
**Q507**	**តើជាទម្លាប់អ្នកស្លៀកខោអាវវែងៗនៅពេលថ្ងៃ ញឹកញាប់ កម្រិតណាក្នុង ១ សប្តាហ៍ ?**	**ចំនួនថ្ងៃស្លៀកពាក់ខោអាវវែង ក្នុង 1 សប្តាហ៍ |____|**			

លរ/រ. No.	សំណួរ QUESTION:	ប្រភេទលេខកូដ CODING CATEGORIES		រំលង Skip
	How often do you wear long sleeves/long pants during the day time?	day/week		
**Q508**	**តើអ្នកប្រើធូកមូសពេលថ្ងៃញឹកញាប់កម្រិតណា?** How often do you use mosquito coils during the day time?	**ចំនួនថ្ងៃប្រើធូកមូសពេលថ្ងៃ ក្នុង 1 សប្តាហ៍** |____| day/week		
**Q509**	**តើអ្នកគិតថា បរិស្ថានក្នុងសហគមន៍ និងជុំវិញផ្ទះរបស់អ្នកស្អាត ដែរ ឬ ទេ?** Do you think your community and household's environment keep clean?	**ទេ** No	0	
		**បាទ/ចា** Yes	1	
		**មិនដឹង** Don't Know	99	
**Q510**	**តើអ្នកគិតថា សម្លៀកបំពាក់ក្នុងផ្ទះរបស់អ្នករៀបចំបានត្រឹមត្រូវដែរ ឬទេ ? (សង្កេត)** Do you think the cloths in your household keep tidy?	**ទេ** No	0	
		**បាទ/ចា** Yes	1	
		**មិនដឹង** Don't Know	99	

បញ្ចប់បទសំភាសន៍ END INTERVIEW
សូមថ្លែងអំណរគុណដល់អ្នកដែលបានចំណាយពេលចូលរួមក្នុងការសំភាសន៍នេះ។

Thank respondent for taking the time to be interviewed.
