# Supplementary material for: Dengue knowledge, attitudes and practices and their impact on community-based vector control in rural Cambodia
Source: PLoS Negl Trop Dis. 2018 Feb 16;12(2):e0006268. doi: 10.1371/journal.pntd.0006268 (PMC5833285; doi:10.1371/journal.pntd.0006268)
Supplement: S2 Survey — (PDF) [file pntd.0006268.s003.pdf]

Container Form for Collection of Larvae/Pupae

Village	name		code		Date		DD		MM		YY	
Household (HH)	name		code		Collector 1 name							
					Collector 2 name							
					Supervisor name							

Fill in field

Container category		Container IDs	Total	Number of containers		Number of containers	
				positive	negative	In	Out
Drum (D)	Fish						
	None						
Water storage jar: small, medium, large (J)	Fish						
	PPF						
	None						
Concrete tank (CT)	Fish						
	None						
Cement basin (CB)	Fish						
	None						
Small pot, <10 L (P)	Fish						
	PPF						
	None						
Flower vase/pot/ tray (F)	Fish						
	PPF						
	None						
Tyre (T)	Fish						
	PPF						
	None						
Can/bottle (C)	Fish						
	None						
Miscellanious -Domestic Use (M)	Fish						
	PPF						
	None						
Others (O)	Fish						
	PPF						
	None						

Container Characterisation Form

				Date	DD	MM	YY
Village	name		code	Collector 1 name			
Household	name		code	Collector 2 name			
				Supervisor name			

Fill in new forms for >4 containers per household [use a new form(s) for more than 4 containers] - (all units in cm)

Container category		Container IDs	Total container	Container no. 1				Container no. 2				Container no. 3				Container no. 4			
				Diameter	Length X Width	Height	Water	Diameter	Length X Width	Height	Water	Diameter	Length X Width	Height	Water	Diameter	Length X Width	Height	Water
Drum (D)	Fish																		
	None																		
Water storage jar (J)	Fish																		
	PPF																		
	None																		
Concrete tank (CT)	Fish																		
	None																		
Cement basin (CB)	Fish																		
	None																		
Small pot, <10 L (P)	Fish																		
	PPF																		
	None																		
Flower vase/tray (F)	Fish																		
	PPF																		
	None																		
Tyre (T)	Fish																		
	PPF																		
	None																		
Can/bottle (C)	Fish																		
	None																		
Miscellanious - Domestic Use (M)	Fish																		
	PPF																		
	None																		
Others (O)	Fish																		
	PPF																		
	None																		

Indoor Resting Adult Summary Form

Village	name		code	

Date		DD		MM		YY
Collector 1 name						
Collector 2 name						
Supervisor name						

Fill in field

House ID	No, people sleep in HH last night	Name of HH	Cup IDs	Adult Mosquitoes		Comments
				Present	Absent	
